# Data structures for compound promiscuity analysis: promiscuity cliffs, pathways and promiscuity hubs formed by inhibitors of the human kinome

**DOI:** 10.2144/fsoa-2019-0040

**Published:** 2019-07-25

**Authors:** Filip Miljković, Jürgen Bajorath

**Affiliations:** 1 Department of Life Science Informatics, B-IT, LIMES Program Unit Chemical Biology & Medicinal Chemistry, Rheinische Friedrich-Wilhelms-Universität, Endenicher Allee 19c, D-53115 Bonn, Germany

**Keywords:** compound promiscuity, human kinome coverage, kinase inhibitors, open access data, promiscuity cliff pathways, promiscuity cliffs, promiscuity hubs, structure–promiscuity relationships

## Abstract

**Aim:**

A large collection of promiscuity cliffs (PCs), PC pathways (PCPs) and promiscuity hubs (PHs) formed by inhibitors of human kinases is made freely available.

**Methodology:**

Inhibitor PCs were systematically identified and organized in network representations, from which PCPs were extracted. PH compounds were classified and their neighborhoods analyzed.

**Data & exemplary results:**

Nearly 16,000 PCs covering the human kinome were identified, which yielded more than 600 PC clusters and 8900 PCPs. Moreover, 520 PHs were obtained.

**Limitations & next steps:**

PC and PCP data structures capture structure–promiscuity relationships. Promiscuity assessment is also affected by data sparseness. Given the rapid growth of kinase inhibitor data, the relevance of PC/PCP/PH information for medicinal chemistry and chemical biology applications will further increase.

## Lay abstract

Promiscuity cliffs (PCs) are formed by structurally very similar (analogous) compounds with large differences in the number of targets they are active against. Inhibitors of human kinases are of high interest in drug discovery and so are PCs they form. This is the case because these PCs reveal structural modifications of inhibitors that strongly influence promiscuity (multitarget activity). Sequences of overlapping PCs form pathways that are rich in structure–promiscuity relationship information. PCs and PCPs of inhibitors covering the human kinome have been systematically identified and these data are made freely available as a basis for further investigations.

## Graphical abstract

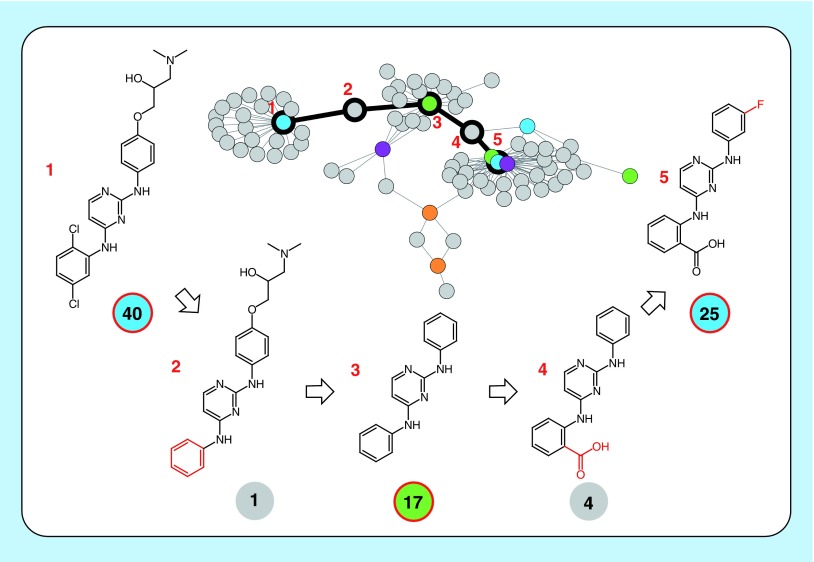

A cluster from a promiscuity cliff (PC) network is shown here in which compounds are represented as nodes and PCs as edges. Nodes are color-coded according to different promiscuity degrees (number of kinase annotations). A PC pathway formed by five kinase inhibitors (1–5) is highlighted (black) and their promiscuity degrees are reported in color-coded circles (red borders indicate promiscuity hubs). Structural modifications distinguishing pairs of compounds along the pathway are colored red.

Compound promiscuity refers to the ability of small molecules to specifically bind to multiple targets [1]. Promiscuity provides the basis for ligand-based polypharmacology [1,2], an emerging concept in drug discovery [2] that represents a departure from the single-target specificity paradigm that has for long dominated drug-discovery efforts [3]. It should be noted that the term promiscuity is often also used with a negative connotation, when referring to compound aggregation- or reactivity-based assay artifacts [4,5]. However, herein promiscuity exclusively refers to genuine multitarget activity of small molecules.

Inhibitors of human kinases are a good example for the interplay between drug polypharmacology and target specificity or selectivity. The efficacy of kinase inhibitor drugs used in oncology clearly depends on multikinase engagement and ensuing polypharmacology [6], whereas the use of kinase inhibitors in other therapeutic areas such as immunology and inflammation or metabolic diseases mostly depends on kinase selectivity [7]. Experimental and computational approaches have been used to analyze promiscuity and selectivity of kinase inhibitors [8–10].

The promiscuity cliff (PC) concept was introduced to aid in the analysis of structure–promiscuity relationships [11,12], in other words, to identify small chemical changes that lead to large apparent differences in promiscuity between structurally analogous compounds. Accordingly, a PC was formally defined as a pair of analogs with a large difference in promiscuity [11]. PCs have been identified among compounds with activity against many therapeutic targets [12] including protein kinases [13]. A large-scale analysis of currently available kinase inhibitors covering more than 80% of the human kinome yielded nearly 16,000 PCs [13].

The formation of PCs can be visualized in network representations where compounds are nodes and edges pairwise PC relationships between nodes [13]. In such networks, PCs form clusters of varying size and complexity. PC clusters are disjoint subgraphs in a PC network. These clusters are rich in structure–promiscuity relationship information, but difficult to analyze. Therefore, as an extension of the PC concept, the PC pathway (PCP) data structure was introduced [13,14]. PCPs are formed in PC clusters and consist of sequences of PCs with overlapping compounds. PCP compounds have alternating high and low promiscuity (or are highly promiscuous and nonpromiscuous). They can be systematically extracted from PC clusters using a computational search method [14]. Nearly 16,000 PCs formed by inhibitors of human kinases were organized in more than 600 separate network clusters [13] and from these clusters, 8900 PCPs were isolated [14]. PCPs often contain so-called promiscuity hubs (PHs). Following network terminology, hubs are densely connected nodes. PHs are highly promiscuous PCP compounds that form large numbers of PCs with weakly or nonpromiscuous structural analogs outside the PCP [14]. PHs also occur in network regions outside PCPs.

PCs, PCPs and PHs provide a wealth of hypotheses for structural determinants of promiscuity and also for additional targets of weakly or nonpromiscuous compounds. For example, structural analogs of a PH might not have been tested against many confirmed PH targets and thus additional targets might be inferred for individual analogs. Taken together, PCs, PCPs and PHs provide valuable information for medicinal chemistry or chemical biology projects. This data note details an open access deposition of PCs identified across the human kinome [13], PCPs extracted from their network clusters [14] and PHs formed by individual kinase inhibitors including clinical compounds. These data are made freely available in an organized and easily accessible form.

## Methodology

### Kinase inhibitor data

Inhibitors of human kinases were collected from several public data sources and activity data were curated [13]. A total of 112,624 unique inhibitors with well-defined activity measurements were obtained that were active against 426 human kinases, corresponding to 82.2% of the human kinome. After removal of potential assay interference compounds [4,5], 105,492 inhibitors remained for PC analysis. For each inhibitor, its promiscuity degree (PD) was calculated as a total number of kinases it was active against.

### Matched molecular pairs

**Figure 1.**
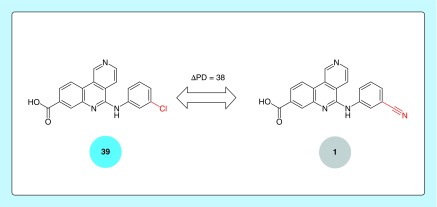
Promiscuity cliff. Shown is an exemplary promiscuity cliff formed by a highly promiscuous and a nonpromiscuous kinase inhibitor. The promiscuity degree of each compound is reported in a color-coded circle. The structural modification distinguishing the cliff compounds is colored red.

For human kinase inhibitors, matched molecular pairs (MMPs) were generated by systematic fragmentation of exocyclic single bonds [15]. An MMP represents a pair of compounds that are only distinguished by a single chemical modification, termed transformation [15]. Transformation size restrictions were introduced as follows [16]: the MMP core shared by two compounds was required to have at least twice the size (number of nonhydrogen atoms) of the transformation substructures. In addition, each substructure was permitted to consist of at most 13 nonhydrogen atoms and their size difference was limited to at most eight atoms. An MMP with these transformation size restrictions represents a pair of structural analogs [16]. An exemplary MMP is shown in Figure 1. The transformation substructures are highlighted in red and the common core structure of the compound (MMP core) is shown in black.

### Promiscuity cliffs

The definition of PCs requires the consideration of two criteria including the *similarity* criterion and *promiscuity difference* criterion. For PC analysis of kinase inhibitors, these criteria were set as follows [13]:

- *Similarity:* formation of transformation size-restricted MMPs.
- *Promiscuity difference:* ΔPD ≥5; PD range of weakly promiscuous MMP inhibitor: [1,4].

We deliberately restrict the PD range of weakly promiscuous compounds in PCs to low promiscuity values (PD <5). This restriction avoids the generation of PC pairs consisting of two highly promiscuous inhibitors (e.g., PD = 40 and PD = 20), which would not be meaningful. Accordingly, this definition ensures that a PC is consistently formed by a highly and weakly or nonpromiscuous (PD = 1) inhibitor. Accordingly, a kinase inhibitor PC with smallest possible PD sum is formed by a pair of inhibitors with PD values of 6 and 1, respectively. Figure 1 shows an exemplary PC.

### Promiscuity cliff pathways

PCPs are defined as linear subgraphs of PC clusters and consist of compounds with alternating high and low promiscuity [13]. From PC network clusters, PCPs are systematically extracted using an algorithm based on breadth-first search for shortest paths [14]. In breadth-first search, edges between neighboring nodes have equal length. Therefore, the shortest path between two nodes is determined as the path containing the smallest number of edges. For visualization, PC clusters in which PCPs are traced are drawn using the Kamada–Kawai force-directed layout algorithm [17].

### Promiscuity hubs

PHs are densely connected nodes in a PC network. For our current analysis, PHs are defined as inhibitors forming at least 10 PCs with structural analogs having a PD value of 1–4 (corresponding to a PH node degree ≥10). As a reference, in the global kinase inhibitor PC network, the mean node degree was approximately 3. We note that PHs may or may not participate in the formation of PCPs. Special attention was paid to kinase inhibitors at different stages of clinical development (clinical kinase inhibitors) [9] that qualified as PHs. Furthermore, PHs are organized into analog series (ASs) using the compound–core relationship algorithm [18]. This MMP-based method systematically extracts ASs with single or multiple substitution sites from compound collections [18]. For compound–core relationship calculations, transformation size-restricted MMPs are applied (as described above).

## Data & exemplary results

### PCs & clusters

**Figure 2.**
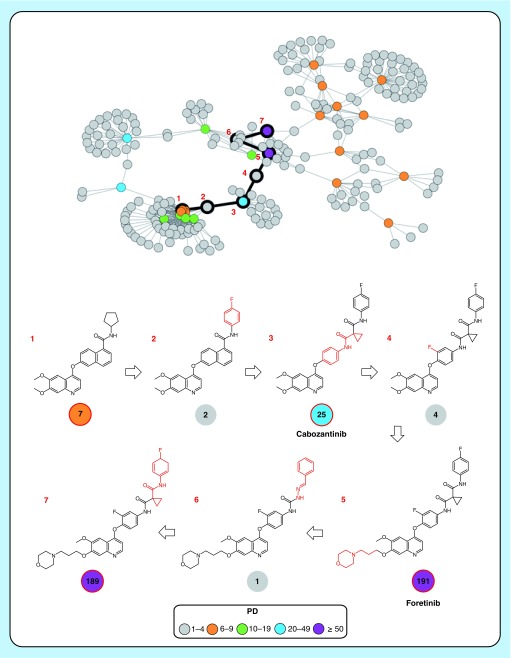
Promiscuity cliff cluster and pathway. Shown is a promiscuity cliff cluster in which a promiscuity cliff pathway formed by seven kinase inhibitors (1–7) is highlighted (black). Nodes are color-coded according to different promiscuity degree values. Structures of promiscuity cliff pathway compounds are shown and their promiscuity degrees are reported in color-coded circles. Structural modifications distinguishing pairs of compounds along the pathway are colored red. Inhibitors 1, 3 (cabozantinib), 5 (foretinib) and 7 are promiscuity hubs. Cabozantinib and foretinib are clinical kinase inhibitors. PD: Promiscuity degree.

The 105,492 kinase inhibitors yielded 15,939 PCs that were formed by 10,741 unique inhibitors including 1653 compounds with PD ≥6. These PCs had ΔPD values ranging from 5 to 294. In a global kinase inhibitor PC network, the 15,939 PCs were organized in 622 separate clusters that contained 2 to 633 inhibitors forming 1 to 1351 PCs [13]. Figure 2 shows an exemplary PC cluster.

### Promiscuity cliff pathways & promiscuity hubs

From the 622 PC clusters, a total of 8900 PCPs were algorithmically extracted via breadth-first search (see above). For further methodological details, the interested reader is referred to the original publication [14]. These PCPs consisted of 3 to 17 nodes. The characteristic feature of PCPs is their sequence of alternating highly and weakly promiscuous (or nonpromiscuous) compounds. For each PCP, the cumulative ΔPD was calculated over all pairs of nodes. These ΔPD values ranged from 10 to 869. In the PC cluster in Figure 2, a PCP is traced that consists of seven inhibitors including clinical kinase inhibitors cabozantinib (compound 3; 25 kinase annotations) and foretinib (compound 5), a pan-kinome inhibitor with 191 kinase annotations.

In the global PC network, a total of 520 inhibitors (4.8%) qualified as PHs on the basis of the criteria given above. Most PCPs (7749; 87.1%) contained at least one PH. The 520 PHs were involved in the formation of 12,131 PCs (76.1%) with 7278 weakly or nonpromiscuous structural analogs. The 12,131 PCs included 6997 PCs that involved 4300 inhibitors having a single kinase annotation. Thus, more than half of the PCs with highly promiscuous PHs involved nonpromiscuous compounds. These findings emphasized the high information content of PH network neighborhoods, revealing many possible structure–promiscuity relationships and suggesting a wealth of kinase target hypotheses for structural analogs of PHs.

### PHs formed by clinical kinase inhibitors

**Figure 3.**
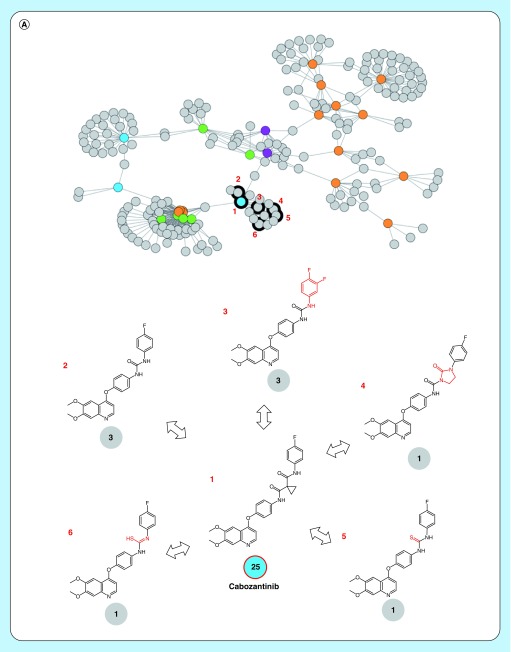

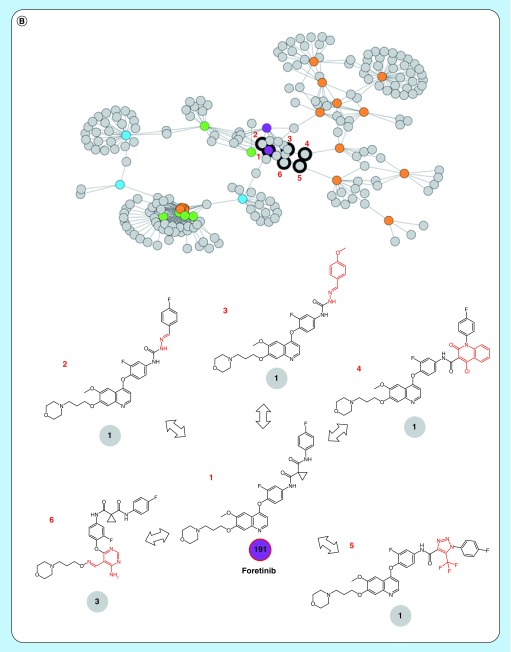
Promiscuity hub neighborhoods. Shown are promiscuity cliffs from the network neighborhoods of **(A)** cabozantinib and **(B)** foretinib. The presentation is according to Figure 2. Exemplary inhibitors forming promiscuity cliffs with the two clinically relevant promiscuity hubs are numbered and their structures are shown.

The 520 PHs contained 68 clinical kinase inhibitors, 37 of which were also found in PCPs. Cabozantinib and foretinib, shown in Figure 2, were two of these clinically relevant PHs occurring in PCPs. Their neighborhoods are depicted in Figure 3A and B, respectively. The 68 clinical PHs formed more than 90% of all PCs involving 129 clinical kinase inhibitors contained in the global network, thus highlighting their central role for promiscuity exploration.

### Analog relationships between PHs

To further explore structural relationships between PHs, we also investigated whether they might form ASs. A subset of 334 of the 520 PHs was found to form 88 ASs that consisted of 2 to 25 analogs. The remaining 186 PHs were not involved in analog relationships. Thus, PHs were not only structurally closely related inhibitors but also included a variety of other compounds. However, for each of these highly promiscuous kinase inhibitors, 10 or more weakly or nonpromiscuous structural analogs were available. Therefore, PHs and their neighborhoods provide many opportunities for experimental follow-up investigations.

### Data deposition

The collection of kinase inhibitor PCs, PCPs and PHs is made available in three separate, tab-delimited text files. In addition, a readme.txt file specifies all entries and abbreviations in the PC, PCP and PH data files.

For each PC, the Simplified Molecular Input Line Entry Systems (SMILES) [19] representation of the inhibitors, SMILES pattern of the transformation, common MMP core, compound identifiers and PD value of the inhibitors are provided.

For each PCP, the pathway identifier, pathway length, list of compounds forming the PCP, available PHs and cumulative ΔPD value are given.

Furthermore, all PHs are listed with their compound identifiers from the PC file and SMILES representations and clinical kinase inhibitors are identified.

The PC, PCP, PH and readme files are provided in an open access deposition on the ZENODO platform [20].

## Limitations & next steps

Data sparseness is likely to affect promiscuity analysis. Sparseness refers to the situation that not all inhibitors might have been extensively tested against all kinases. Importantly, PCs, PCPs and PHs uncover all detectable promiscuity patterns, regardless of whether they reveal structural determinants of promiscuity or provide additional target hypotheses. Thus, these data structures make it possible to further investigate structure–promiscuity relationships and their potential origins in detail. Increasing availability of x-ray structures enables further exploration of PCs. Potential origins of PC formation can be investigated on the basis of protein–ligand interactions taking active site characteristics into consideration. Structural analysis of PCs on a larger scale than currently possible is expected to provide new insights into structural patterns that are responsible for promiscuous versus selective binding events.

Kinase inhibitor data have rapidly grown in recent years, more so than could have been anticipated, and there is no end in sight. Therefore, we will continue to search for PCs, PCPs and PHs and periodically update our collections to further support promiscuity exploration.

**Executive summary**

**Background**

- Compound promiscuity is defined.
- The promiscuity cliff (PC) and promiscuity cliff pathway (PCP) concepts are introduced.
- Promiscuity hubs (PHs) are introduced in the context of PC networks.

**Methodology**

- PC criteria are specified.
- PC cluster and PCP analysis are described.
- PH neighborhoods are discussed.

**Data & exemplary results**

- PC, PCP and PH statistics are reported.
- Exemplary PC clusters, PCPs and PHs are presented.
- Clinical kinase inhibitors forming PHs are analyzed.
- Structural relationships between PHs are systematically detected.
- An open access deposition of PCs, PCPs and PHs is described.

**Limitations & next steps**

- The potential influence of data sparseness on promiscuity is discussed.
- Further growth of kinase inhibitor data is anticipated.
- Data growth motivates continued promiscuity exploration.
